# Feasibility of a novel photoproduction of ^225^Ac and ^227^Th with natural thorium target

**DOI:** 10.1038/s41598-021-04339-9

**Published:** 2022-01-10

**Authors:** Kwangho Ju, Yonghee Kim

**Affiliations:** Department of Nuclear and Quantum Engineering, Korea Advanced Institute of Science and Technology (KAIST), 291 Daehak-ro, Yuseong-gu, Daejeon, 34141 Republic of Korea

**Keywords:** Applied physics, Engineering

## Abstract

We propose an innovative way to produce both ^225^Ac and ^227^Th, two precious radioisotopes enabling promising targeted alpha therapy, in a natural thorium target bombarded with a 30–90 MeV electron beam. Bremsstrahlung photons in the target are analyzed by MCNP and in-situ photonuclear transmutation of ^232^Th is evaluated by using the TENDL nuclear data. In the photo-transmutation analysis, 13 nuclides including ^229^Th and ^231^Pa are modelled. Special procedures with chemical separations are also proposed to produce pure ^225^Ac and ^227^Th in separate streams. In addition, performance of the new approach is compared with conventional methods in terms of the ^225^Ac and ^227^Th yields. After a Th target is bombarded with a 500 kW electron beam for a year, yearly ^225^Ac yield is ~ 8.47 GBq (semi-permanently) and yearly ^227^Th yield is ~ 48.9 GBq over 50 years, and their yields are at least doubled in a 2-year irradiation. This work will help increase global supply of the two precious isotopes and would invariably help advance TAT-related researches and developments.

## Introduction

Targeted alpha (*α*) therapy (TAT) is becoming a promising way to treat incurable cancers^1^. An *α*-emitter conjugated with a suitable carrier can transfer lethal energy to cancer cells precisely even when the tumor tissue is tiny. TAT minimizes damages in normal tissues due to high linear energy transfer (60–230 keV/μm) and short range (50–90 μm) of *α*-particles in human body. Demand for *α*-emitters has been increasing since the US FDA approved ^223^Ra chloride as a radiopharmaceutical. For cancer treatments, both ^225^Ac and ^227^Th are regarded as best *α*-emitters due to an adequate half-life and emission of multiple *α*-particles by daughter nuclides. After an outstanding efficacy was reported for ^225^Ac in treating prostate cancers, a lot of researches on TAT with ^225^Ac are underway globally. Meanwhile, ^227^Th is also getting lots of attention due to its chemical affinity to be easily bonded with various antibodies. In particular, ^227^Th decays to ^223^Ra, which is an officially approved *α*-emitter for clinical use, and it is rather free from regulatory issues other than its daughter^2–4^. Radiopharmaceuticals labelled with ^227^Th are being tested for various cancer treatments as well^2,5^.

Despite of the high potential of ^225^Ac and ^227^Th, lack of their supply is impeding progress in radiopharmaceutical for TAT. Even for ^225^Ac, the annual supply is only about one thirtieth of its demand that reached about 1850 GBq in 2019^6^. The only commercial way to produce ^225^Ac is a milking from ^229^Th cow which is a decay product of the legacy ^233^U stockpile^6,7^. It is known that the stockpile was mainly produced in the Th-fuelled nuclear reactors in the 1960s in US and Russia^8,9^. Consequently, the ^225^Ac supply is rather monopolized and affordable ways are strongly required for progress in the ^225^Ac-based TAT.

Researches are currently underway to find out alternative methods to produce ^225^Ac. For example, the Tri-lab project team is looking into ways to directly extract ^225^Ac from a proton-irradiated thorium target, and produced 81.4 GBq of ^225^Ac in a 10-day irradiation of 200 MeV proton beam at 165 μA^10,11^. Regarding this method, one concern is ~ 0.3% yield of ^227^Ac contaminating ^225^Ac in addition to the relatively high proton energy required. It is known that ^227^Ac may cause severe level of radiation dose for the rest of patient’s lives if it is injected with ^225^Ac^12^. Meanwhile, utilizing ^226^Ra(p,2n)^225^Ac reaction is regarded as the most feasible approach due to a low proton energy. Institute for Transuranium Elements (ITU) produced ~ 485 MBq of ^225^Ac by irradiating a RaCl_2_ target with a 28 MeV beam for 45.3 h at 50 μA beam current^7,13^. However, it is known that procurement and treatment of the highly radioactive Ra target are challenging and costly. Though other methods including irradiation in nuclear reactors have been investigated to utilize ^232^Th(n,γ)^233^U and ^226^Ra(n,2n)^225^Ra reactions, low yield of ^225^Ac and ^227^Ac impurity catches up with their commercialization^7^.

The situation is even worse in the case of ^227^Th production. The natural ^227^Ac yield from ^235^U is very small due to long half-life of ^235^U and ^227^Th briefly exists as a daughter nuclide of ^227^Ac. Currently, there are no other options but to produce ^227^Ac from the ^226^Ra(n,γ)^227^Ra reaction^2,14^, which means that the aforementioned Ra target issue remains unresolved and a costly neutron source is required.

In response to the high demand of the ‘rarest drugs’ ^225^Ac and ^227^Th, we propose an innovative method to semi-permanently produce the *α*-emitters with an electron accelerator and natural thorium (^232^Th) target. The Monte Carlo N-Particle transport (MCNP) version 6.2 code is used to calculate X-ray or photon generation and heat deposition in the Th target^15^. The photo-transmutations of ^232^Th into ^225^Ac or ^227^Th are estimated by using the TALYS-generated Evaluated Nuclear Data Libraries (TENDL) cross-sections for various (*γ*,xn) photonuclear reactions^16^.

## Methods

### Photonuclear reactions in thorium target

**Figure 1:**
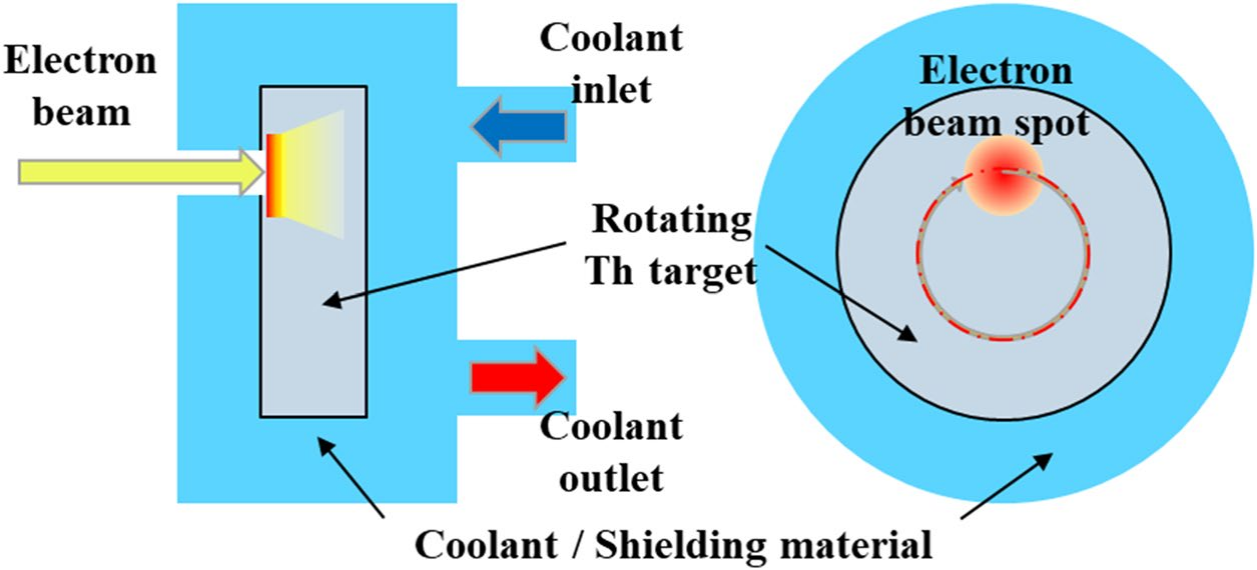
Schematic diagram of thorium target system.

Figure 1 shows schematic diagram of the proposed Th target system for photo-production of both ^225^Ac and ^227^Th. A metallic cylindrical Th target is directly bombarded by an electron beam to generate Bremsstrahlung photons and trigger photonuclear reactions. The target should rotate to manage locally-deposited heat during beam irradiation and it is supposed to be equipped with a cooling and radiation protection system as in the TRIUMF Isotope Separator and Accelerator (ISAC) facility^17^. Taking into account target cooling, the electron beam power is set to 500 kW and a relatively large target is considered (radius = 10 cm and thickness = 6 cm) to minimize the photon leakage from the target. To achieve appropriate target cooling, we adopt a beam diameter of 40 mm and a rotational speed of 120 RPM (rotation per minute). Detailed discussion on the heat deposition and removal is provided in Supplementary Information Sect. A.

**Figure 2:**
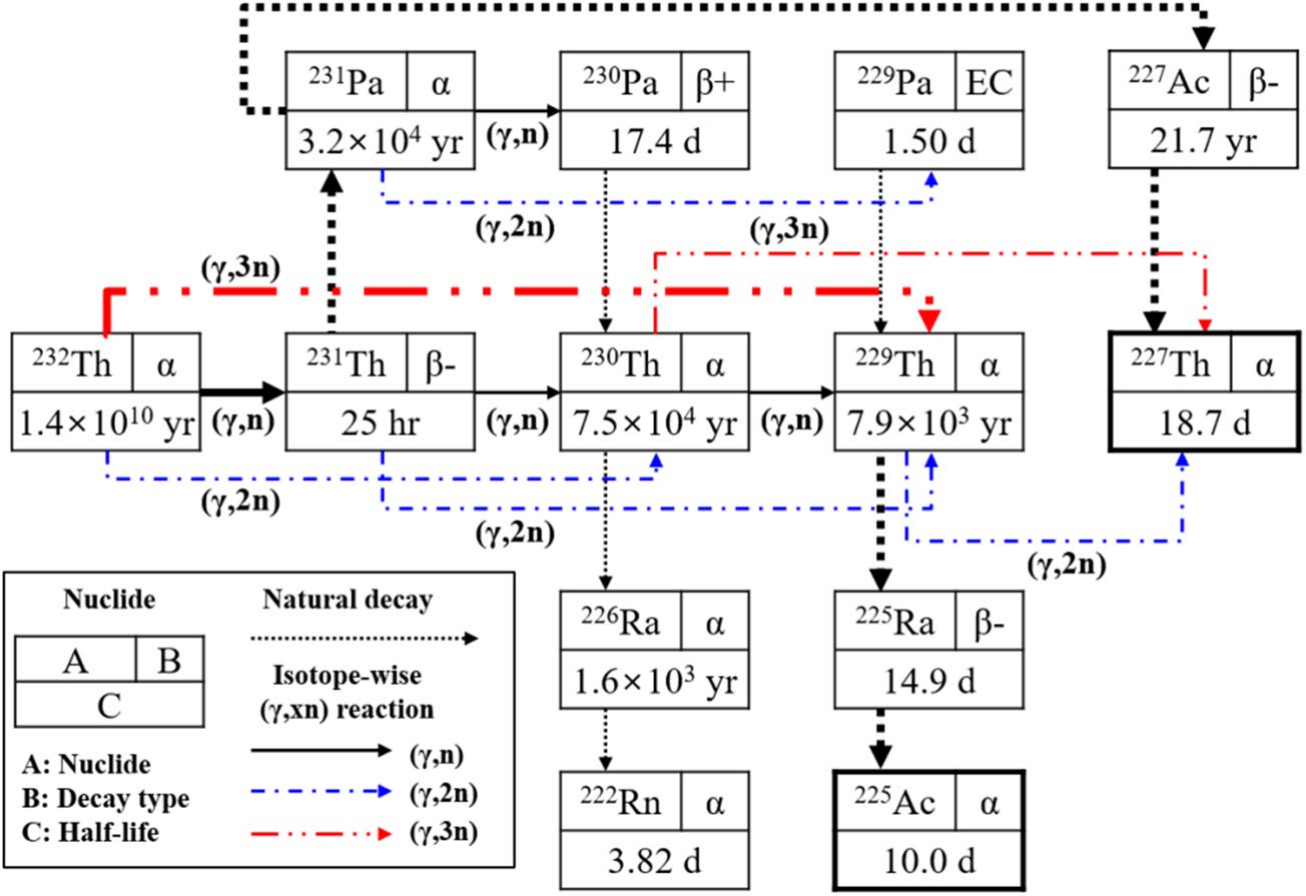
Photonulcear reactions and decays in Th target during electron beam irradiation.

Figure 2 describes major photonuclear reactions and transmutation chains of 13 nuclides in the Th target. Two most significant pathways to generate ^225^Ac and ^227^Th are highlighted with bold lines respectively: ^232^Th(γ,3n)^229^Th → ^225^Ra → ^225^Ac and ^232^Th(γ,n)^231^Th → ^231^ Pa → ^227^Ac → ^227^Th. Isotopic yields involving multiple photonuclear reactions with other thorium isotopes are actually very small. Other actinium isotopes can also be generated from (γ,p) and (γ,np) photonuclear reactions. However, they quickly decay to thorium isotopes except for ^229^Th(γ,np)^227^Ac reaction, which is insignificant due to very small (γ,np) cross-section. We also neglect the photo-fission of ^232^Th, in spite of noticeable probability, since both fission products and neutrons hardly affect yield of ^225^Ac and ^227^Th. Meanwhile, the impact of photo-fissions on heat deposition turns out to be noticeable and it is discussed in Supplementary Information Sect. A. In the Th target, many fast neutrons are also generated by the photonuclear reactions including the photo-fissions. When the Th target is bombarded with 500 kW electron beam (70 MeV energy and 7.14 mA current), the neutron production is roughly estimated to be ~ 1.94E + 15 #/s. However, it is found that most of the fast neutrons (> 97%) simply escape the target and cannot contribute to the production of ^225^Ac and ^227^Th.

**Figure 3:**
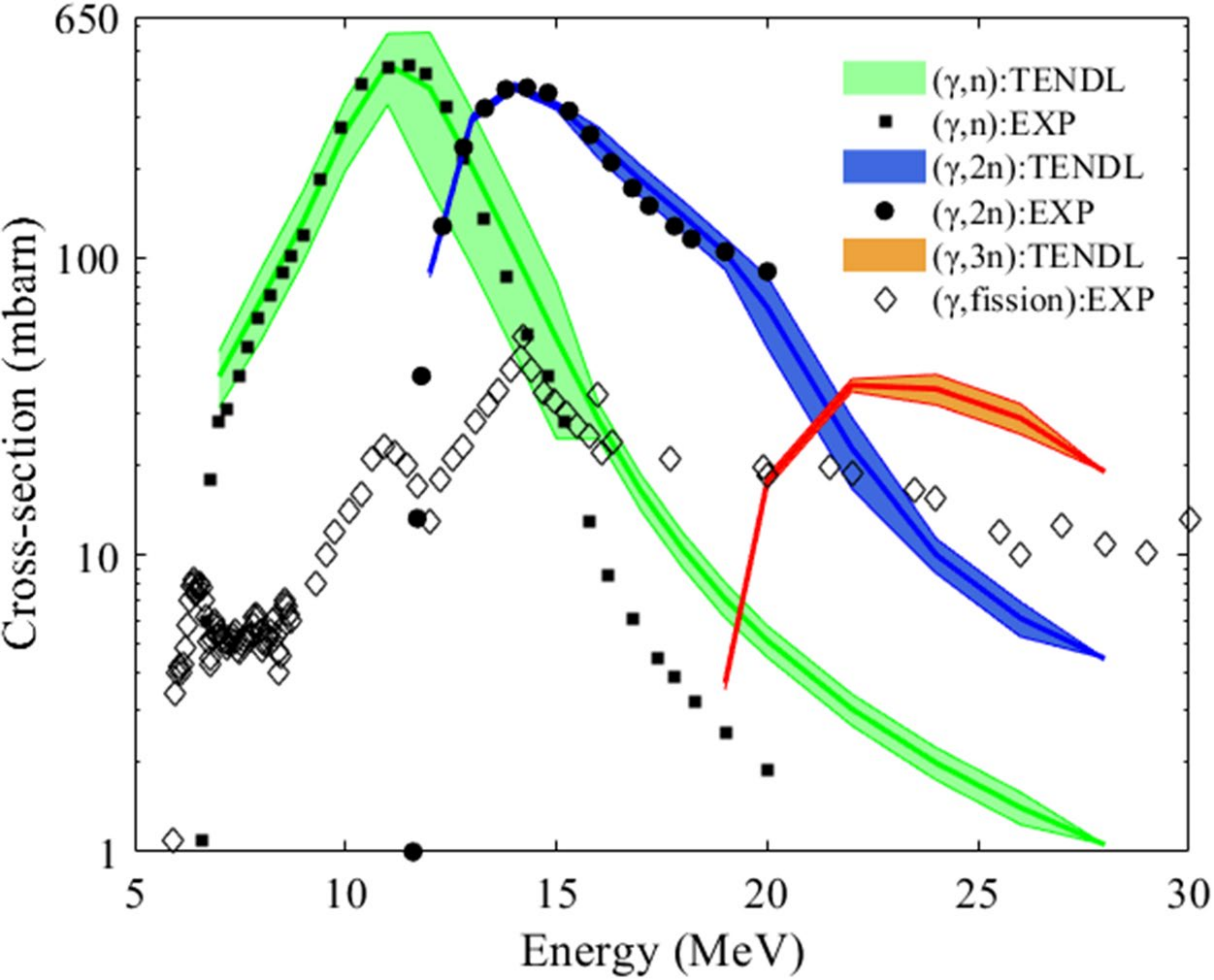
Comparison between TENDL and experimental data for (γ,xn) cross-sections of ^232^Th.

Measured cross-sections of the photonuclear reactions of the Th and Pa isotopes are unknown except for (γ,n) and (γ,2n) reactions of ^232^Th^18^. In order to calculate the photonuclear transmutation rate, the TENDL nuclear data are utilized. Figure 3 shows experimental (γ,xn) and photo-fission cross-sections of ^232^Th together with the TENDL data for comparison. Except for overrated TENDL data of (γ,n) cross-section at a high energy tail, most of the experimental data for (γ,n) and (γ,2n) reactions are within one standard deviation of the TENDL data. While nuclear reaction modelling codes including TALYS generally provide rather uncertain parameters for photonuclear reactions, it is known that the parameter of giant dipole resonance (GDR) reactions such as (γ,xn) of heavy nuclides is slowly varying with the atomic mass number and the deviation from one nuclide to another is rather small^19–21^. Therefore, a GDR cross-section of a nuclide can be well predicted based on the experimental data of neighboring nuclei. Based on the measured cross-sections and general credibility of the TENDL library as shown in Fig. 3, actual cross-sections of ^232^Th(γ,3n)^229^Th and other (γ,xn) reactions with similar mass number such as ^231^Pa are expected to be similar to the TENDL data. Impacts of TENDL data uncertainty on the isotopic yields are given in Supplementary Information Sect. E.

As indicated in Fig. 3, high energy (> 6 MeV) photons are required for the photonuclear (γ,xn) reactions of ^232^Th and the electron energy should exceed about 30 MeV. The Bremsstrahlung photon source in the Th target is generated by using the well-validated MCNP code. For a given photon source, number density of ‘A’ nuclide (*N*^*A*^) is estimated by solving the following balance equations.

1 $$ \frac{{dN^{A} }}{dt} = - \lambda_{Nat}^{A} N^{A} - \lambda_{\gamma }^{A} N^{A} + \lambda_{Nat}^{Other} N^{Other} + \lambda_{{(\gamma ,{\text{x}}n)}}^{Other} N^{Other} , \, $$

2 $$ \lambda_{{(\gamma ,{\text{x}}n)}}^{A} = \left( {\int {\sigma_{{(\gamma ,{\text{x}}n)}}^{A} \frac{{dN_{\gamma } }}{dE}dE} } \right), \, \lambda_{\gamma }^{A} = \sum\limits_{{{\text{x}} = 1}}^{3} {\lambda_{{(\gamma ,{\text{x}}n)}}^{A} , \, } $$

where *λ*_*Nat*_ is natural decay constant, *λ*_*(γ,*x*n)*_ is an effective decay constant for a (γ,xn) reaction, *λ*_*γ*_ is summation of the effective decay constants for all (γ,xn) reactions, and *dN*_*γ*_*/dE* is the photon spectral density obtained by MCNP 6.2. A space-dependent photon spectral density should be used for accurate evaluation of the isotopic yields. The last two terms in Eq. (1) denote production of ‘*A*’ nuclide due to decay or transmutation of other nuclides. Detailed coupled equations for the 13 nuclides in Fig. 2 are described in Supplementary Information Sect. B. Spatial distribution of the photon source in Eq. (2) is pre-evaluated with MCNP and it is assumed to be constant since transmutation rate of ^232^Th is extremely low in the rotating Th target.

### Procedures to retrieve pure ^227^Th and ^225^Ac

**Figure 4:**
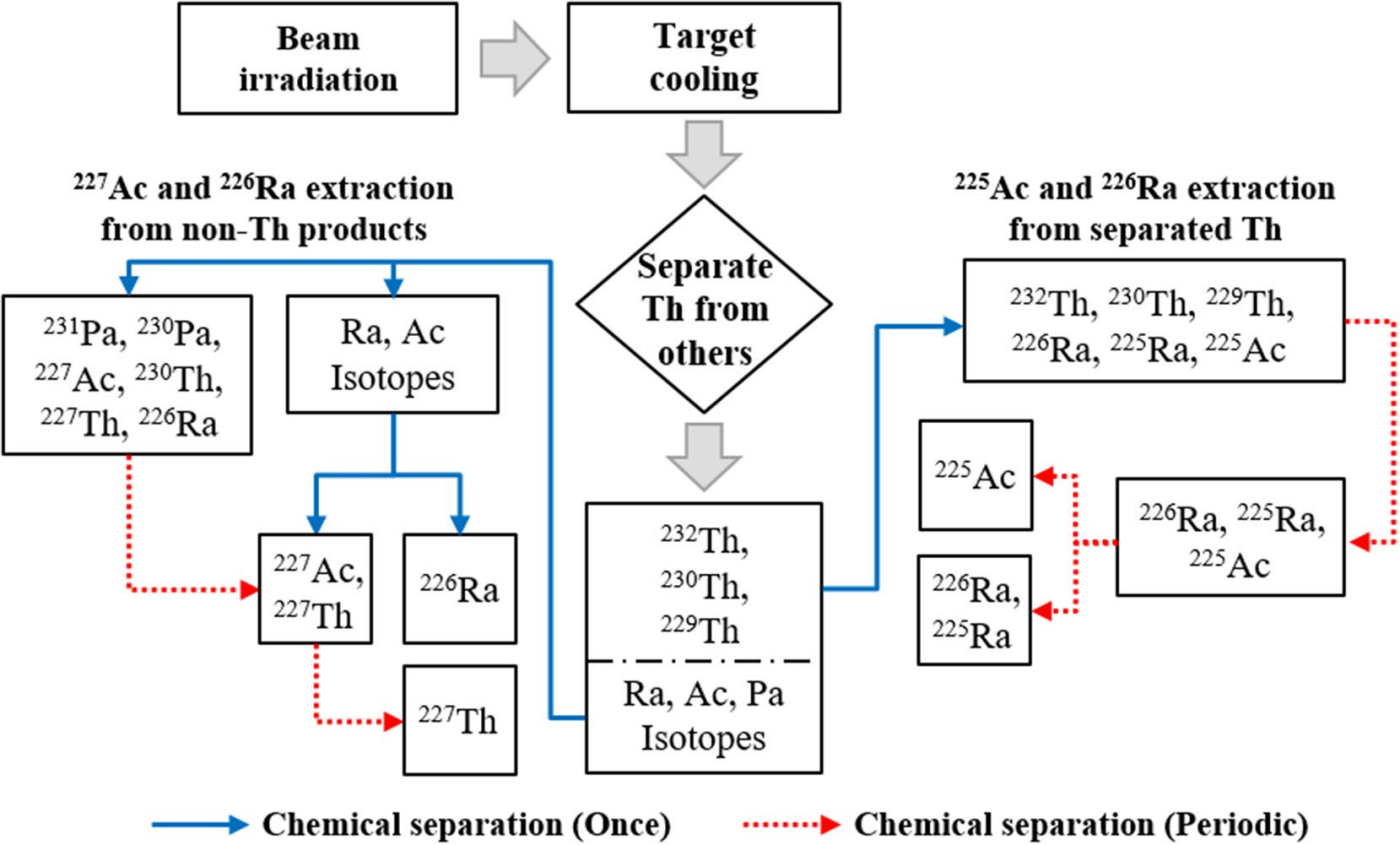
Procedure for semi-permanent milking of ^225^Ac and ^227^Th.

To produce pure ^225^Ac and ^227^Th from the irradiated target, their parent nuclides, ^229^Th and ^227^Ac, should be separated in order to avoid the ^227^Ac contamination. There have been a few experimental validations for extraction of ^225^Ac from ^233^U or ^229^Th stockpiles^22,23^. For the same purpose, we propose a simple strategy and chemical processings for periodic milking of the *α*-emitters, as shown in Fig. 4.

Purpose of the cooling phase is to let ^231^Th (progenitor of ^227^Ac) completely decay out. After irradiation, the target should be cooled for a certain time to minimize the ^227^Ac impurity for pure ^225^Ac extraction. Once ^231^Th disappears by its natural decay, all remaining Th isotopes are chemically separated from the others, then ^225^Ac is purely produced by decay of ^229^Th and this chemical extraction of ^225^Ac can be near permanent due to long half-life of ^229^Th. It should be noted that ^226^Ra can be also produced due to decay of ^230^Th in the separated Th stream and it can be used as the target material for other accelerated-based ^225^Ac production methods.

Meanwhile, pure ^227^Th can be chemically extracted from the separated irradiation byproducts, which are basically Ra, Ac, and Pa isotopes, i.e., ^231^Pa slowly decays to ^227^Ac decaying again to ^227^Th with a half-life of ~ 22 years. If ^227^Th is directly extracted from the separated byproducts without any additional chemical partitionings, the ^227^Th can be blended with ^230^Th generated by a natural decay of ^230^Pa, which may cause an unwanted hazard induced by its daughter nuclides. For the purity of ^227^Th, we chemically partition the byproducts into three parts: Ra, Ac, and Pa isotopes (blue lines in the non-Th products in Fig. 4). Then, ^227^Ac is extracted from the separated Pa and moved to the actinium part where pure ^227^Th can be purely extracted periodically. It is important to note that ^227^Th production is quite semi-permanent (over 50 years) since half-life of ^227^Ac is about 22 years. All chemical procedures in Fig. 4 are very analogous with ones used in the current ^225^Ac extraction from natural thorium target bombarded by proton beam^24,25^, and they are considered to be readily available. It is also obvious that the ^225^Ac and ^227^Th yields should increase if the amount of ^229^Th and ^231^ Pa in the target is increased with a prolonged beam irradiation and/or higher electron current.

## Results

### Parameter optimization for electron beam

A 500 kW electron beam is considered to evaluate the feasibility and performance of the new photo-production of ^225^Ac and ^227^Th. In order to find optimal parameters for the 500 kW electron beam, isotopic yield of ^229^Th and ^231^Pa (parent nuclei of ^225^Ac and ^227^Th) is evaluated for different beam diameters and electron energies. For accurate evaluation of the photon spatial distribution, the target is subdivided into small zones in both radial and axial directions, which is described in detail in Supplementary Information Sect. C.

**Figure 5:**
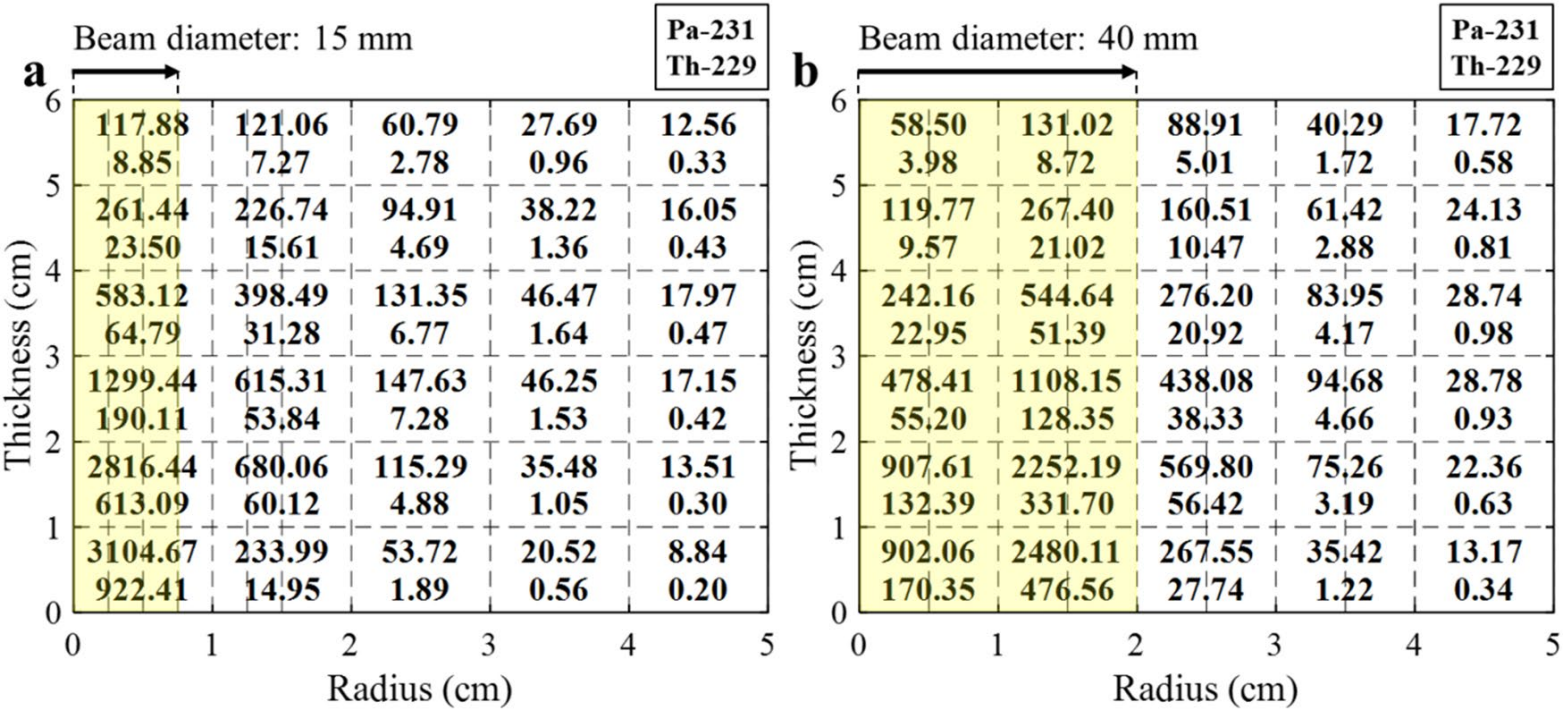
Isotopic yield (MBq) in a fixed target with different beam diameters.

For the sensitivity analysis, it is assumed that a 70 MeV beam bombards a fixed target with a 5 cm radius and a 6 cm height. The target size is large enough to minimize the leakage of Bremsstrahlung photons from the target. Figure 5 shows the spatial distribution of isotopic yields with different electron beam sizes (yellow area) after a one-year beam irradiation and cooling of a month. The spatial isotopic yield is evaluated with a different spatial discretization of the target depending on the beam size, as indicated by the dotted lines in Fig. 5. One notes that total yield of ^229^Th drops by ~ 22% when beam diameter is increased from 15 to 40 mm, whereas the 40 mm beam enhances ^231^Pa production slightly. The clearly lower ^229^Th yield with the 40 mm beam is because contribution of the ^231^Pa(γ,2n)^229^Pa reaction can be noticeably lowered due to lower concentration of ^231^Pa with a larger beam diameter. Meanwhile, the slightly higher ^231^Pa production for the 40 mm beam is simply due to the slower depletion rate of ^232^Th in a bigger irradiated region. It is clear that a smaller beam size is favorable in terms of the ^229^Th yield if the integrity of the target is guaranteed during irradiation.

**Figure 6:**
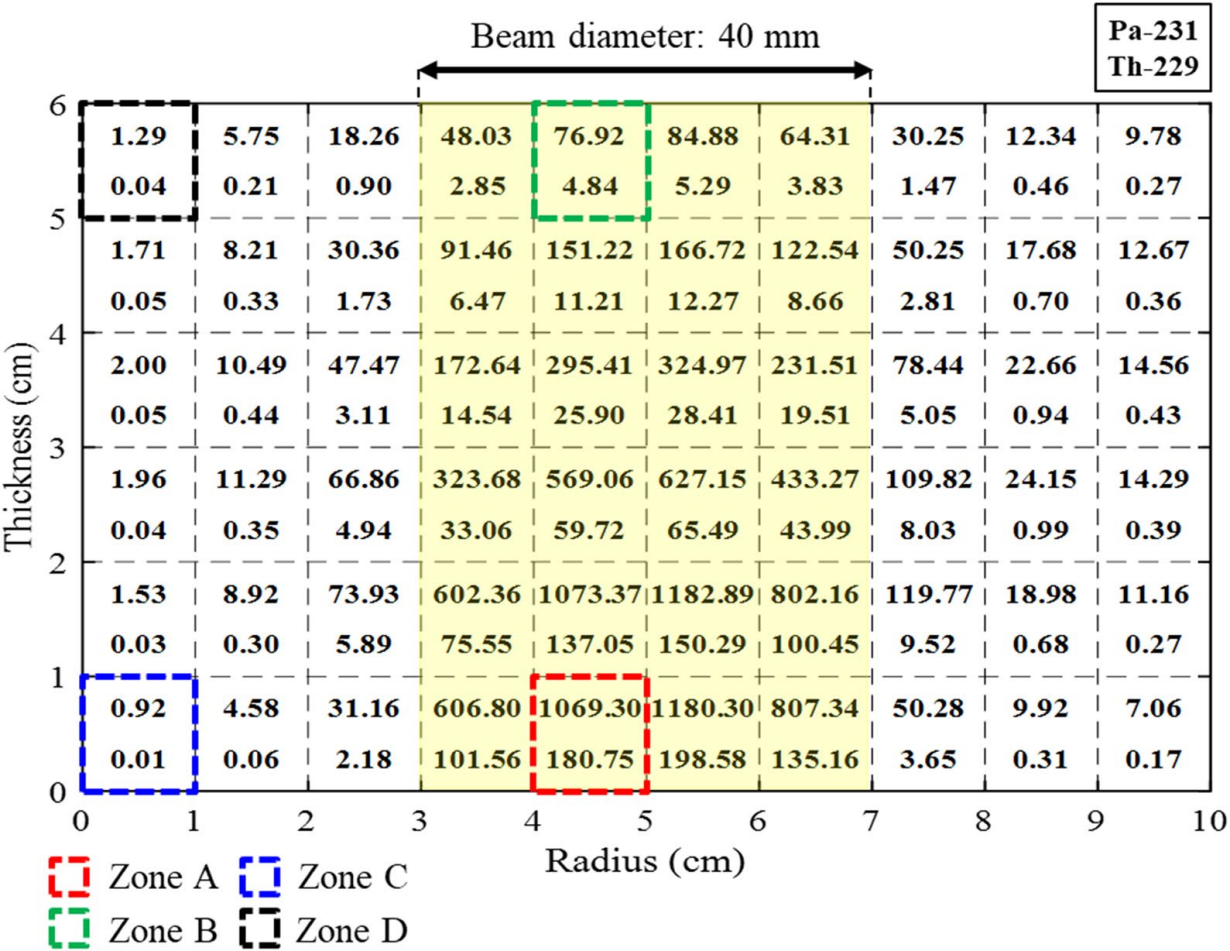
Isotopic yield (MBq) in the rotating target with 40 mm beam diameter.

In the actual rotating Th target as shown in Fig. 1, the effective beam size or irradiated area is significantly increased compared with a fixed target. In the Th target with a 120 RPM speed, it is assumed that the spatial photon spectral density is uniform in the azimuthal direction and they are evaluated with a detailed discretization in the radial direction. The isotopic yields of ^229^Th and ^231^ Pa are given in Fig. 6 when the target radius is 10 cm and beam diameter is 40 mm. It is noted that total yield of ^229^Th is slightly lower than that of the fixed target due to further dispersion of the electron beam, and production of ^231^ Pa is slightly increased. The isotopic yields in the rotating target are compared with ones in the fixed target in Table 1.

**Table 1:** Isotopic yield (GBq) with different beam irradiation conditions.

Beam diameter (target condition)	15 mm (fix)	40 mm (fix)	40 mm (rotating)
^229^Th	2.04	1.59	1.48
^231^ Pa	11.36	11.82	12.05

**Figure 7:**
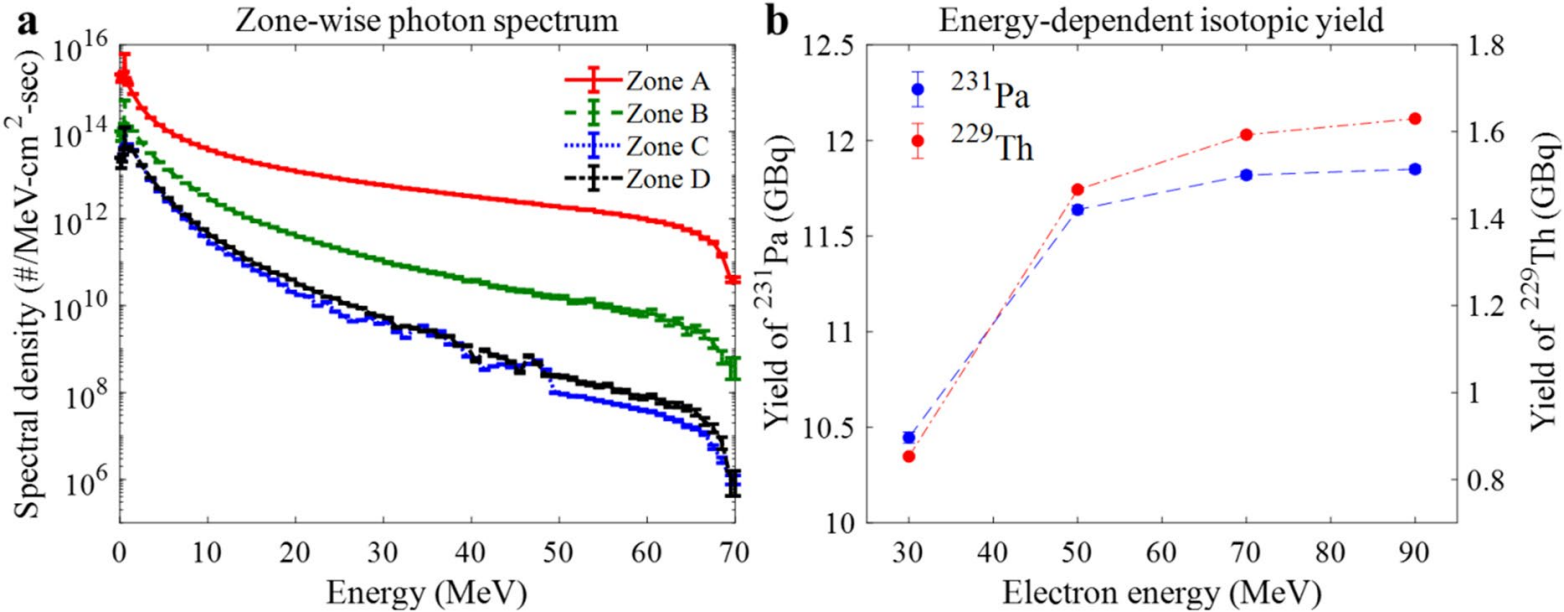
Zone-wise photon spectral densities for 70 MeV electron beam and isotopic yield of ^229^Th and ^231^ Pa as a function of electron energy.

Figure 7a shows MCNP-evaluated zone-wise photon spectral densities for the rotational target in Fig. 6. One clearly notes that the photon flux is highest in the zone A and decreases more quickly in the radial direction due to a localized electron distribution. The highly position-dependent photon density results in the space-dependent yield distribution in Fig. 6. The number of electrons in the MCNP simulation is 8,000,000, leading to accurate photon spectral density in the whole target region. It is mentioned that impact of uncertainty of the photon flux on the *α*-emitter yield is less than 0.6%.

Figure 7b shows the isotopic yield of ^229^Th (source of ^225^Ac) and ^231^Pa (source of ^227^Th) in the whole target as a function of the electron energy after a one-year beam irradiation and cooling of a month. The yield for both isotopes converges to an asymptotic value with electron energy since the photonuclear cross-sections of ^232^Th are maximized below 30 MeV as indicated in Fig. 3. The optimal energy is about 70 MeV for the ^231^Pa, while it is slightly above 90 MeV for ^229^Th. This is because ^229^Th production is mainly dominated by the (γ,3n) reaction requiring higher electron energy than in the (γ,n)-dominating ^231^Pa production. Based on the results, 70 MeV is considered to be near optimal to produce the two isotopes simultaneously as incremental yield of ^229^Th is marginal above 70 MeV.

### Periodic yield of ^225^Ac and ^227^Th

**Figure 8:**
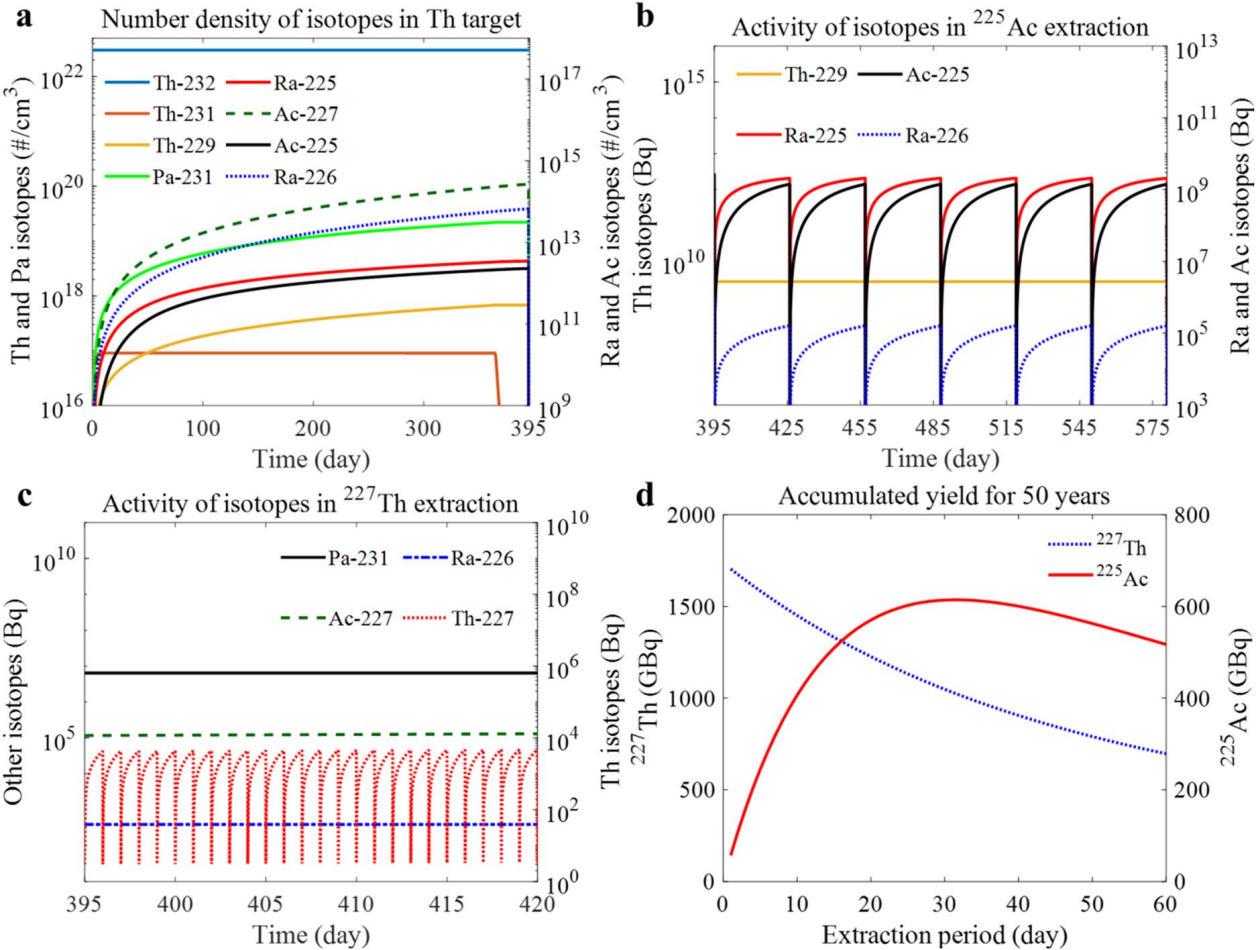
Isotopic evolution in Th target, periodic extraction and accumulated yield of ^225^Ac and ^227^Th.

Figure 8 shows time-dependent isotope-wise number density in the rotating target and activity of important nuclides in the periodic extractions. The ^227^Ac can be less than 10^–8^% in the separated Th stream after 30-day cooling as shown in Fig. 8a. Nuclides inventories in the separated Th and non-Th products for periodic extractions of ^225^Ac and ^227^Th are shown in Fig. 8b,c. Long-term extraction of both α-emitters can be realized due to a sufficient amount of their parent nuclei and it is clear that extraction of pure ^225^Ac and ^227^Th is possible in each separated stream. Optimal extraction or milking period for ^225^Ac and ^227^Th is determined so that accumulated yield should be maximized, as shown in Fig. 8d.

When ^225^Ac is extracted for 50 years after a one-year beam irradiation, its accumulated yield increases until 31-day cooling period and gradually dwindles. Meanwhile, there is no local maximum point of the accumulated ^227^Th yield with different extraction periods due to a large difference of half-life between ^227^Th and its parent nuclide, and basically the shorter the extraction, the better in terms of the accumulated yield. In this work, we regard 1 day for ^227^Th and 31 days for ^225^Ac as optimum extraction period.

**Figure 9:**
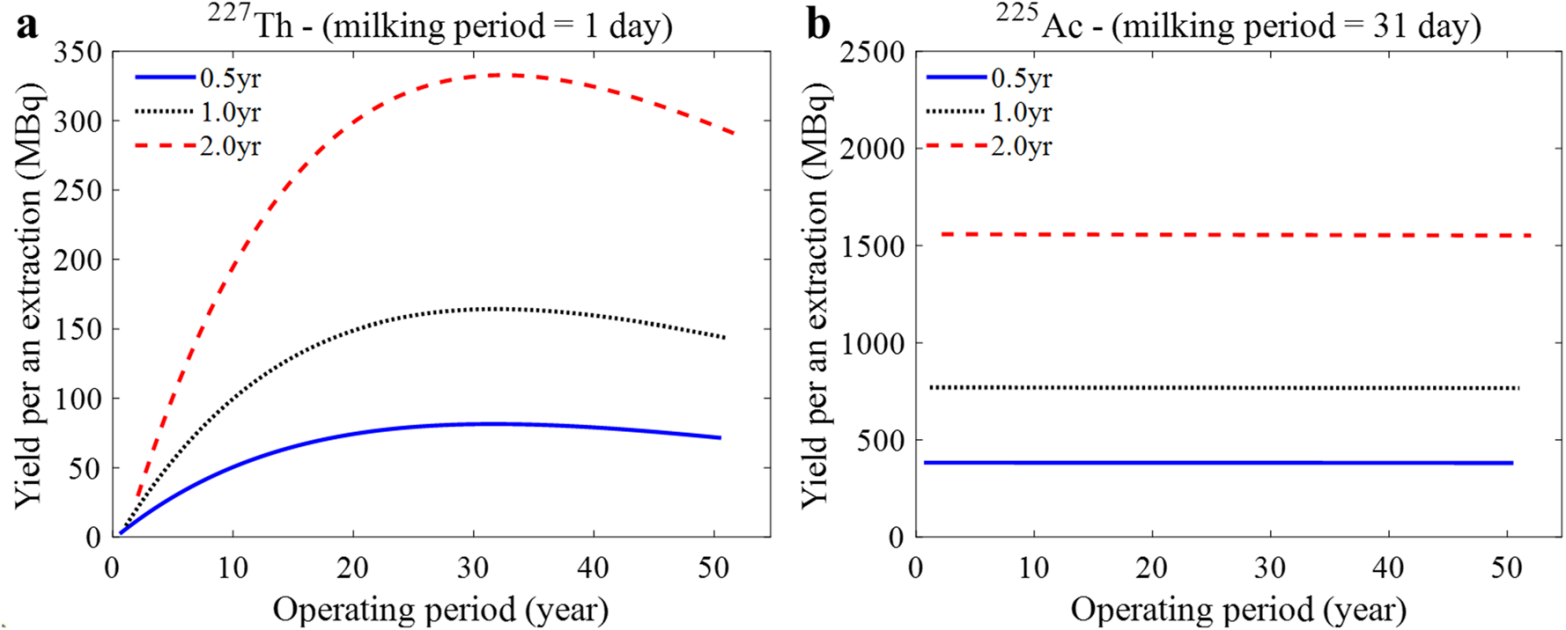
Yield of ^225^Ac and ^227^Th per extraction with different beam irradiation times.

Figure 9 shows the yield of ^225^Ac and ^227^Th per an extraction for three irradiation periods with a 70 MeV beam over a 50-year operation. One observes in Fig. 8 that ^225^Ac yield is almost constant, while the ^227^Th yield increases over 30 years and then slowly decreases due to the longer half-life of ^227^Ac than that of ^225^Ra decaying to ^225^Ac, which is the root cause of the low ^227^Th yield right after irradiation. If a daily ^227^Th yield is too small, a longer extraction period can be adopted in the early operational period (See Supplementary Information Sect. D).

### Discussion

Table 2 compares the proposed method with other approaches using a ^226^Ra target to produce ^225^Ac and ^227^Th. Performances of the new method are based on the mean TENDL data. Their variations due to uncertain TENDL data are discussed in Supplementary Information Sect. E . Regarding the existing methods, an optimistic ^225^Ac yield is also evaluated by improving beam and target parameters^13,26,27^. For an optimistic ^227^Th yield of the reactor-based approach^14^, an optimal irradiation time and a relatively large target are considered (see Supplementary Information Sect. F).

**Table 2:** Comparison of ^225^Ac and ^227^Th production methods.

Facility		Nuclear reactor		Proton accelerator		Electron accelerator		
Governing reaction		^226^Ra(n,γ)^227^Ra		^226^Ra(p,2n)^225^Ac		^226^Ra(γ,n)^225^Ra		This work
Case		Exp^1^	Potential^2^	Exp^1^	Potential^2^	Exp^1^	Potential^2^	Potential^2^
Neutron flux or beam current		1.09 × 10^14^ n/cm^2^-s		50 μA	500 μA	26 μA		7.14 mA
Irradiation time (day)		11.7	540	1.89	0.042	0.121		365
Particle energy		< 10 keV		28 MeV		18 MeV		70 MeV
Target mass		5.1 μg	5 g	30 mg	210 mg	40 mg	2.52 g	22 kg
Yield per irradiation (MBq)	^225^Ac	–	–	484.7	743.7	1.07	67.7	Single irradiation
	^227^Th	Single irradiation		–	–	–	–	Single irradiation
Yearly yield (GBq)	^225^Ac	–	–	74.1	501.1	0.63	39.8	8.47 (17.1)^4^
	^227^Th^3^	0.0004	1440	–	–	–	–	48.9 (100.1)^4^

^1^Experiment.

^2^Optimistic expectation.

^3^50-year average yield.

^4^2-year irradiation.

The new method requires only one ‘long’ irradiation and ^225^Ac can be extracted semi-permanently. However, a ‘short’ irradiation is always necessary for each production of ^225^Ac in the others. In order to compare a yearly yield, it is assumed, based on engineering judgements, that there should be at least 12-h preparation time between irradiations in the conventional methods. The yearly ^227^Th yield is averaged over a 50-year operation since it is time-dependent in both new and reactor-based approaches.

The optimistic reactor-based ^227^Th yield is much higher than that of the new method due to the very favorable target and irradiation conditions. Unfortunately, the nuclear reactor is extremely costly and is not available in many countries. Although the yearly ^225^Ac yield of the new method is lower than the optimistic expectation of the others, the following advantages should be recalled: (1) pure ^225^Ac can be produced in the new photo-production, while there should be some level of ^227^Ac contamination in methods utilizing a Th target bombarded by a proton beam, (2) a semi-permanent source is produced after a single irradiation in a compact electron accelerator, (3) both ^225^Ac and ^227^Th are simultaneously produced. It is also noteworthy that both yields are rather proportional to the irradiation time since contribution of multiple photonuclear reactions to the yields is quite marginal in the rotational Th target.

## Conclusions

The newly proposed ^225^Ac and ^227^Th photo-production method is deemed to be viable and promising in several aspects. First of all, it can produce pure ^225^Ac and ^227^Th by irradiating a ‘cheap’ natural Th target with a 50–70 MeV electron beam and then using the conventional chemical procedures. The electron energy needs to be over 50 MeV and 70 MeV turns out to be optimal for the application. To maximize the ^225^Ac and ^227^Th yield, a rotational Th target is required and it is shown that yearly yield of ^225^Ac and ^227^Th can be ~ 8.5 GBq and 49 GBq, respectively, for a 500 kW beam. The required electron accelerator is easily available and the new method can get all counties to benefit from TAT. More importantly ^225^Ac production is almost permanent and ^227^Th can be produced much over 50 years after a beam irradiation. Yearly ^227^Th yield is about four times higher than that of ^225^Ac, and the new method is particularly useful for production of emerging ^227^Th. We believe that this work will help increase global supply of the two precious isotopes and would invariably help advance TAT-related researches and developments.

Taking into account the photonuclear cross-sections, the expected ^227^Th yield seems to be rather reliable while the ^225^Ac yield is more uncertain due to absence of measurement data for the governing (γ,3n) cross-section of ^232^Th. Therefore, the ^232^Th(γ,3n)^229^Th cross-section should be experimentally evaluated for more concrete conclusions.

## Supplementary Information

Supplementary Information.
