# Supplementary material for: Feasibility of a novel photoproduction of 225Ac and 227Th with natural thorium target
Source: Sci Rep. 2022 Jan 10;12:372. doi: 10.1038/s41598-021-04339-9 (PMC8748787; doi:10.1038/s41598-021-04339-9)
Supplement: Supplementary file 1 — Supplementary Information. [file 41598_2021_4339_MOESM1_ESM.docx]

# Supplementary information

1. **Conceptual design of the Th target**

When the Th target is bombarded by a 500 kW electron beam, most of the energy is deposited in the target and a special cooling system should be in place. For 70 MeV electrons uniformly distributed in a circular bunch, heat deposition in a cylindrical target is analyzed by MCNP 6.2. Total heat deposited in the target is ~466 kW and about 4.3% of the total heat is generated by the photo-fission. In the MCNP analysis, the maximum uncertainty of the heat source is ~4.2 % in the lowest source region. Figs. A1a and A1b show heat densities for two beam diameters, 15 mm and 40 mm, without taking into account target’s rotation. For the 15 mm beam diameter, the maximum heat density is about 139 J/mm^3^ just beneath the bombarded surface, which is far beyond thorium’s vaporization enthalpy (26.8 J/mm^3^). Although the heat density can be reduced to 20.4 J/mm^3^ by increasing the diameter up to 40 mm as shown in Fig. A1b, it is not tolerable in terms of target’s integrity.

**
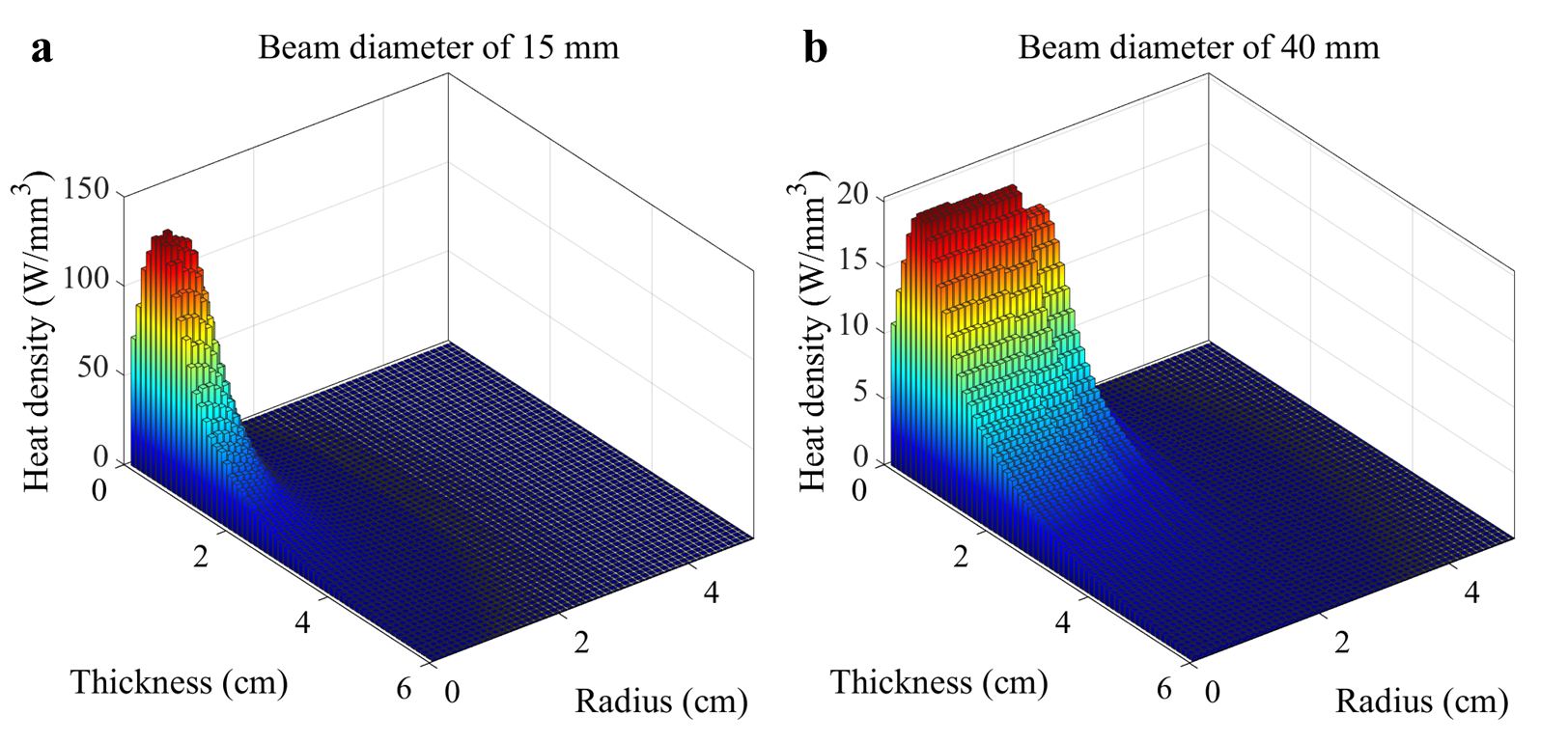
**

Figure A1. Volumetric heat distribution in a fixed Th target.

We believe that total heat in the Th target can be well removed by adopting the cooling system used in Isotope Separator and Accelerator (ISAC) in TRIUMF. The key idea of the cooling system is to disperse electron beam by rotating the target. Figure A2 shows heat densities in a rotating target for the two beam diameters. The maximum heat densities can be reduced a lot to 6.5 J/mm^3^ and 3.5 J/mm^3^, respectively.

**
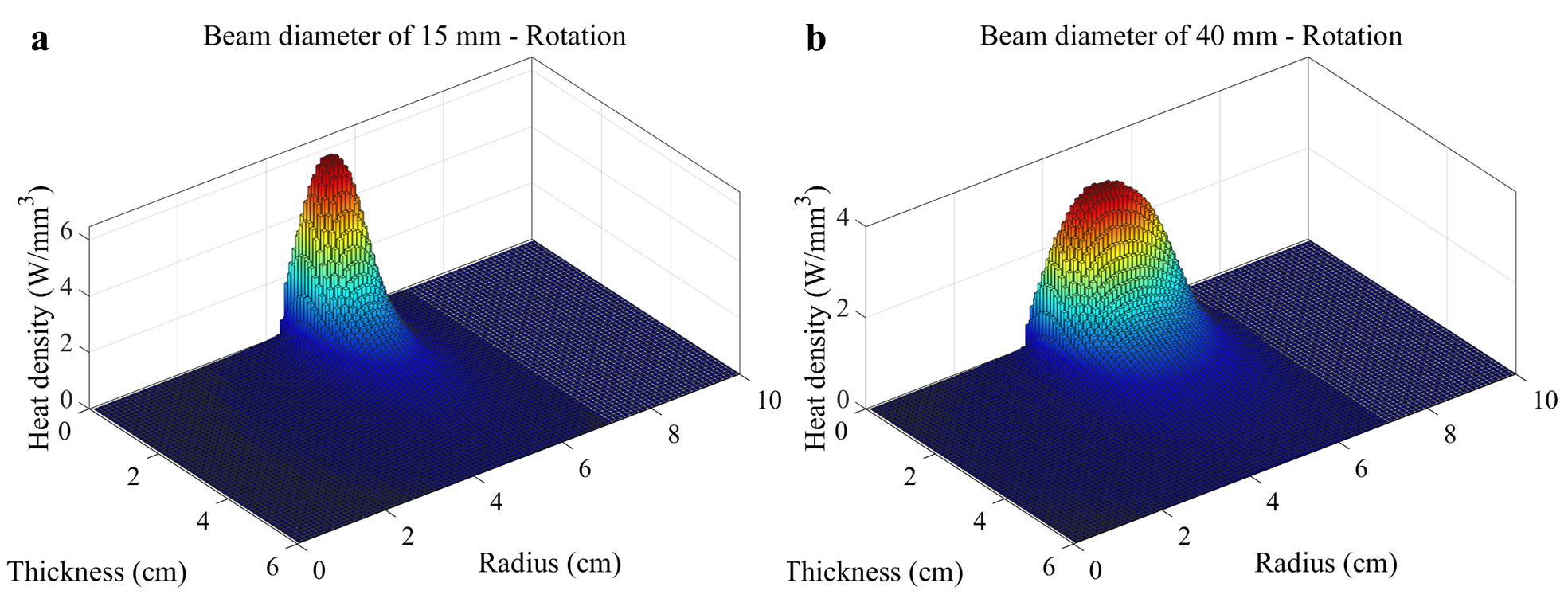
**

Figure A2. Volumetric heat distribution in a rotating Th target.

In the ISAC apparatus, a total of 315 kW heat from a 500 kW electron beam is successfully removed by a water coolant even when the peak heat density is quite higher (187 W/mm^3^) than that of the current Th target. Though the total heat in the current Th target is higher, it is expected that the cooling system design is feasible with optimization of the rotational speed, beam size, beam position and coolant conditions such as flow rate and speed. Based on the ISAC design, pre-conceptual analyses were done to evaluate feasibility of the rotating Th target system in this work using the COMSOL 5.6 Multiphysics software^1^. Figure A3a depicts a conceptual design of the Th target system, where a 40 mm diameter beam is impinging on an annulus Th target (radial thickness=10 cm, axial thickness=6 cm) cladded with a 0.2 mm Ta layer. Distance between the target and beam centers is 92.5 mm. A hull holds a rotating target with a 60 mm diameter central hole where a spinning shaft is placed. Target’s rotation should be clockwise and the speed is determined to be 120 RPM (rotation per minute) to minimize the target temperature and the coolant flow rate is 4.5 kg/sec, resulting in a small temperature rise of 0.5 K at exit. There is a 45 mm thick water layer on the top and bottom of the target and a 20 mm thick water on the side. Thickness of water layer right below a 1 mm thick beam window is only 4 mm to minimize interactions with the incident electron beam. A graphite beam window is temporally adopted in this work as in the ISAC design. A steady-state water flow pattern on the top surface of the spinning target is given in Fig. A3b.


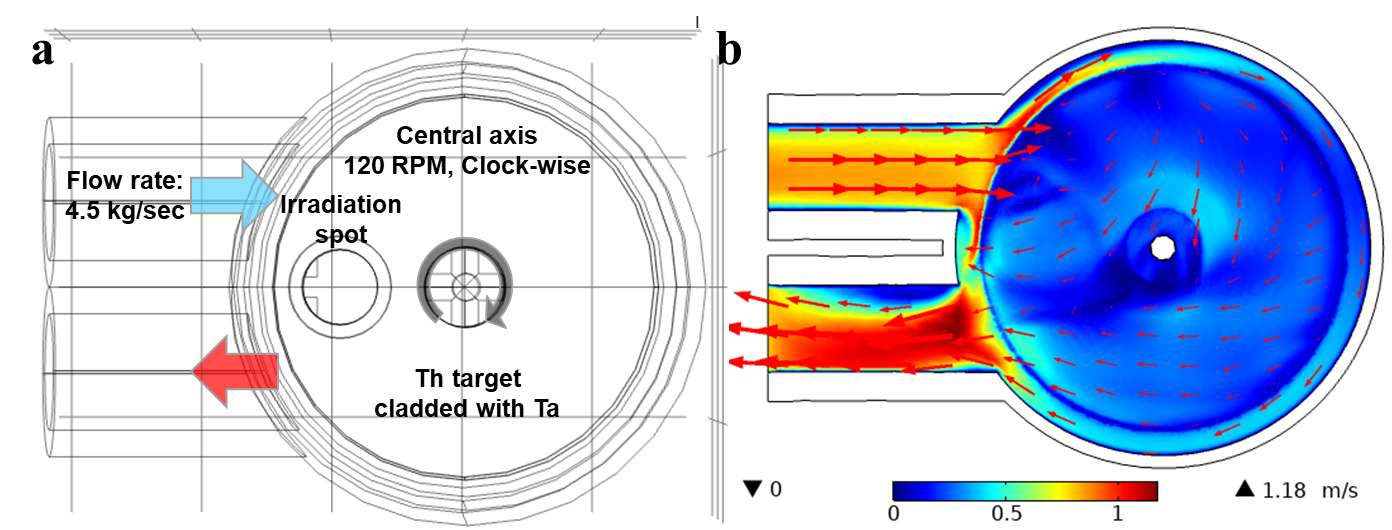


Figure A3. Configuration of Th target system and coolant velocity field^1^.
(COMSOL 5.6 Multiphysics software, https://www.comsol.com/release/5.6)

Figure A4a shows a hot-spot temperature distribution at steady state, which is strongly dependent on the coolant flow conditions on the irradiated surface of the target. It is observed that partial melting of the Th target happens as indicated in Fig. A4b. Although the target locally melts, it should be noted that the melting region is quite small and fully encapsulated by the Ta clad which has a very high melting temperature of 3,290 K.


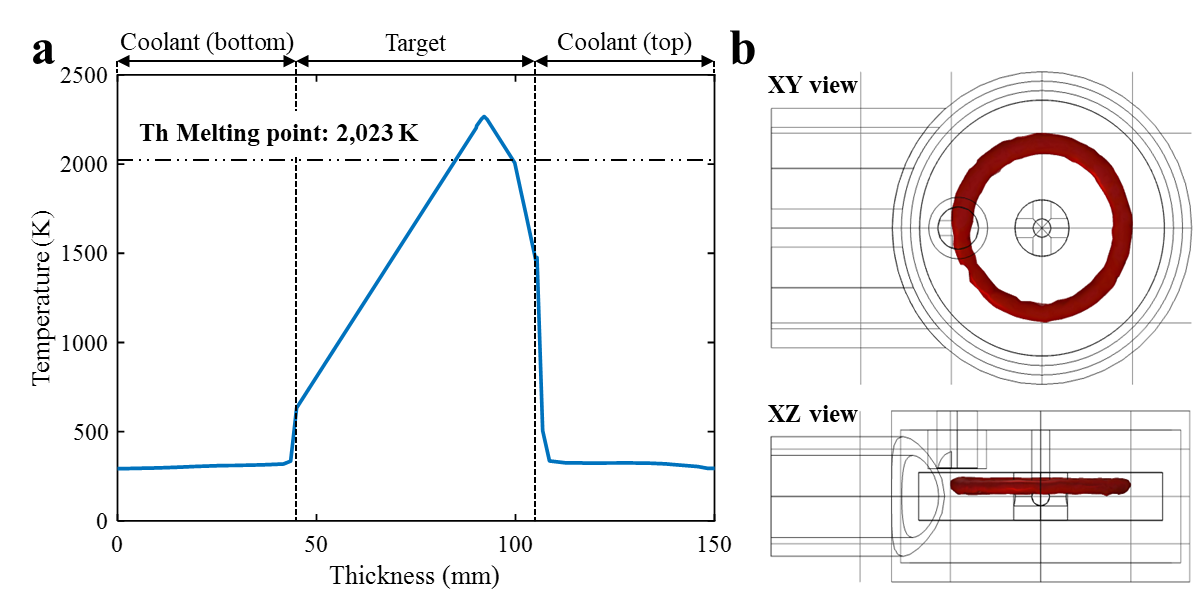


Figure A4. Temperature at steady state and partially melting region in the Th target^1^.
(COMSOL 5.6 Multiphysics software, https://www.comsol.com/release/5.6)

The time-dependent thermal-hydraulics analysis for the rotating Th target system is very challenging and the application of the COMSOL package was rather limited. We believe that a much more optimistic design could be devised with advanced designs tools by experts. It is obviously expected that a bigger beam diameter would prevent the target melting, as indicated in Table A1. If the beam diameter is 60 mm, the maximum target temperature can be lower than Th’s melting point.

Table A1. Maximum target temperature with different electron beam diameters

| Beam diameter (mm) | 15 | 30 | 40 | 50 | 60 |
| --- | --- | --- | --- | --- | --- |
| Maximum temperature (K) | 3,070 | 2,650 | 2,290 | 2,160 | 2,020 |

* Thorium’s melting temperature: 2,023 K

1. Thirteen-nuclide modelling for the nuclear transmutations

In order to estimate the number density of nuclides in the Th target during the whole operational phases, the following equations about 13 nuclides are considered.

$\frac{dN^{^{232}Th}}{dt}=-\left(\lambda_{Nat}^{^{232}Th}+\lambda_{eff}^{^{232}Th}\right)N^{^{232}Th},$ Eq. 1-1

$\frac{dN^{^{231}Th}}{dt}=-\left(\lambda_{Nat}^{^{231}Th}+\lambda_{eff}^{^{231}Th}\right)N^{^{231}Th}+\lambda_{(g,n)}^{^{232}Th}N^{^{232}Th},$ Eq. 1-2

$\frac{dN^{^{230}Th}}{dt}=-\left(\lambda_{Nat}^{^{230}Th}+\lambda_{eff}^{^{230}Th}\right)N^{^{230}Th}+\lambda_{(g,n)}^{^{231}Th}N^{^{231}Th}+\lambda_{(g,2n)}^{^{232}Th}N^{^{232}Th}+0.916\lambda_{Nat}^{^{231}Pa}N^{^{231}Pa},$ Eq. 1-3

$\begin{matrix} \frac{dN^{^{229}Th}}{dt}=-\left(\lambda_{Nat}^{^{229}Th}+\lambda_{eff}^{^{229}Th}\right)N^{^{229}Th}+\lambda_{(g,n)}^{^{230}Th}N^{^{230}Th}+\lambda_{(g,2n)}^{^{231}Th}N^{^{231}Th} \\ \text{ }+\lambda_{(g,3n)}^{^{232}Th}N^{^{232}Th}+0.995\lambda_{Nat}^{^{229}Pa}N^{^{229}Pa}, \end{matrix}$ Eq. 1-4

$\frac{dN^{^{231}Pa}}{dt}=-\left(\lambda_{Nat}^{^{231}Pa}+\lambda_{eff}^{^{231}Pa}\right)N^{^{231}Pa}+\lambda_{Nat}^{^{231}Th}N^{^{231}Th},$ Eq. 1-5

$\frac{dN^{^{230}Pa}}{dt}=-\lambda_{Nat}^{^{230}Pa}N^{^{230}Pa}+\lambda_{(g,n)}^{^{231}Pa}N^{^{231}Pa},$ Eq. 1-6

$\frac{dN^{^{229}Pa}}{dt}=-\lambda_{Nat}^{^{229}Pa}N^{^{229}Pa}+\lambda_{(g,2n)}^{^{231}Pa}N^{^{231}Pa},$ Eq. 1-7

$\frac{dN^{^{227}Ac}}{dt}=-\lambda_{Nat}^{^{227}Ac}N^{^{227}Ac}+\lambda_{Nat}^{^{231}Th}N^{^{231}Th},$ Eq. 1-8

$\frac{dN^{^{225}Ac}}{dt}=-\lambda_{Nat}^{^{225}Ac}N^{^{227}Ac}+\lambda_{Nat}^{^{225}Ra}N^{^{225}Ra},$ Eq. 1-9

$\frac{dN^{^{226}Ra}}{dt}=-\lambda_{Nat}^{^{226}Ra}N^{^{226}Ra}+\lambda_{Nat}^{^{230}Th}N^{^{230}Th},$ Eq. 1-10

$\frac{dN^{^{225}Ra}}{dt}=-\lambda_{Nat}^{^{225}Ra}N^{^{225}Ra}+\lambda_{Nat}^{^{229}Th}N^{^{229}Th},$ Eq. 1-11

$\frac{dN^{^{222}Rn}}{dt}=-\lambda_{Nat}^{^{222}Rn}N^{^{222}Rn}+\lambda_{Nat}^{^{226}Ra}N^{^{226}Ra},$ Eq. 1-12

$\frac{dN^{^{227}Th}}{dt}=-\lambda_{Nat}^{^{227}Th}N^{^{227}Th}+\lambda_{Nat}^{^{227}Ac}N^{^{227}Ac},$ Eq. 1-13

where all notations are explained in the Method section. Decay constants *λ_(γ,xn)_* and *λ_eff_* are neglected in the cooling phase and chemical separation period. An implicit Euler method has been used to solve the coupled system of equations in this work.

1. Isotopic yields with different electron beam diameters

When the beam diameter is smaller, Bremsstrahlung photons are getting dense in the irradiated area. Therefore, the spatial distribution of the electron beam can affect concentration of some isotopes generated by (γ,xn) reactions of ^232^Th. Figures C1 to C3 compare isotopic yields of ^229^Th and ^231^Pa with and without taking into account the ^232^Th(γ,3n)^229^Th reaction for different beam diameters. Figure C1a shows best-estimated isotopic yields and the yields without the ^232^Th(γ,3n)^229^Th reaction are given in Fig. C1b for a 40 mm electron beam. Figures C2 and C3 show similar comparison for smaller beam diameters, 30 mm and 15 mm, respectively. Total isotopic yields for the 3 beam sizes and transmutation pathways are summarized in Table C1.


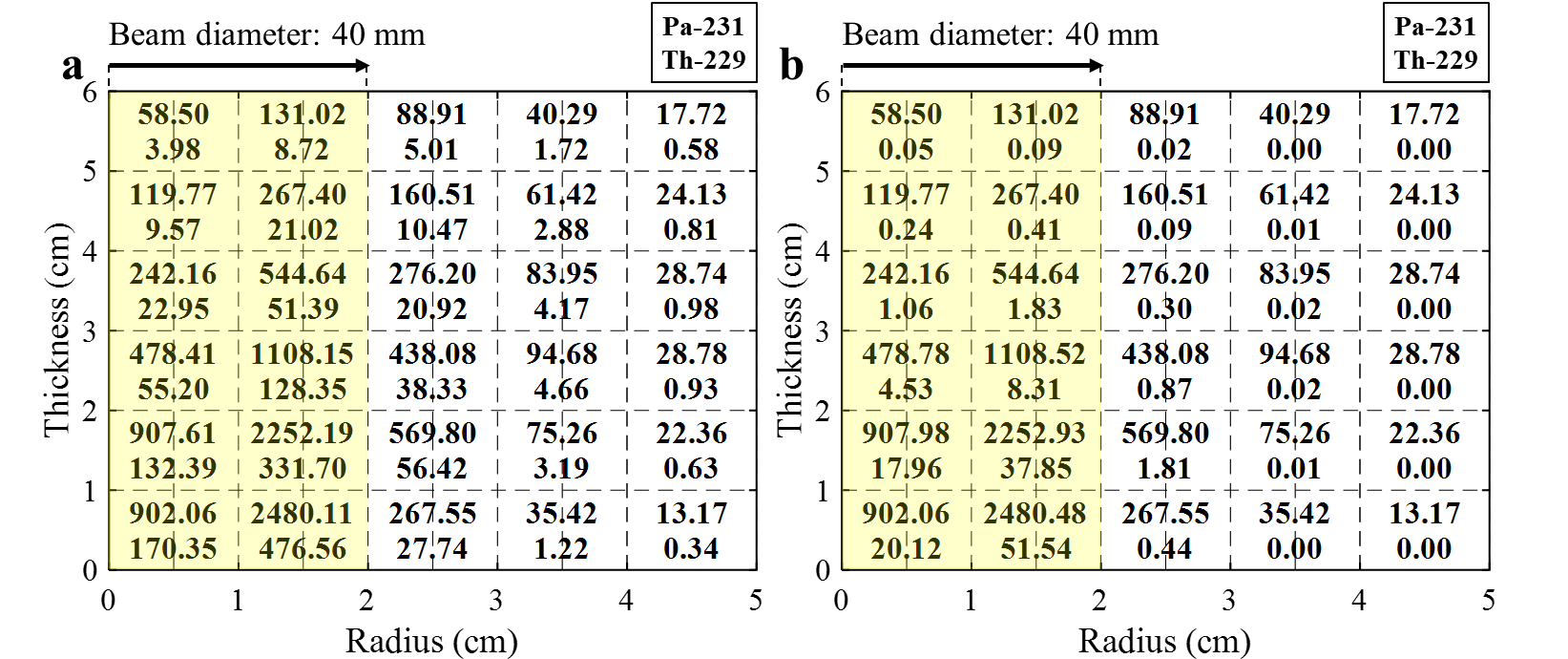


Figure C1. Isotopic yields (MBq) with and without ^232^Th(γ,3n)^229^Th reactions for 40 mm beam diameter


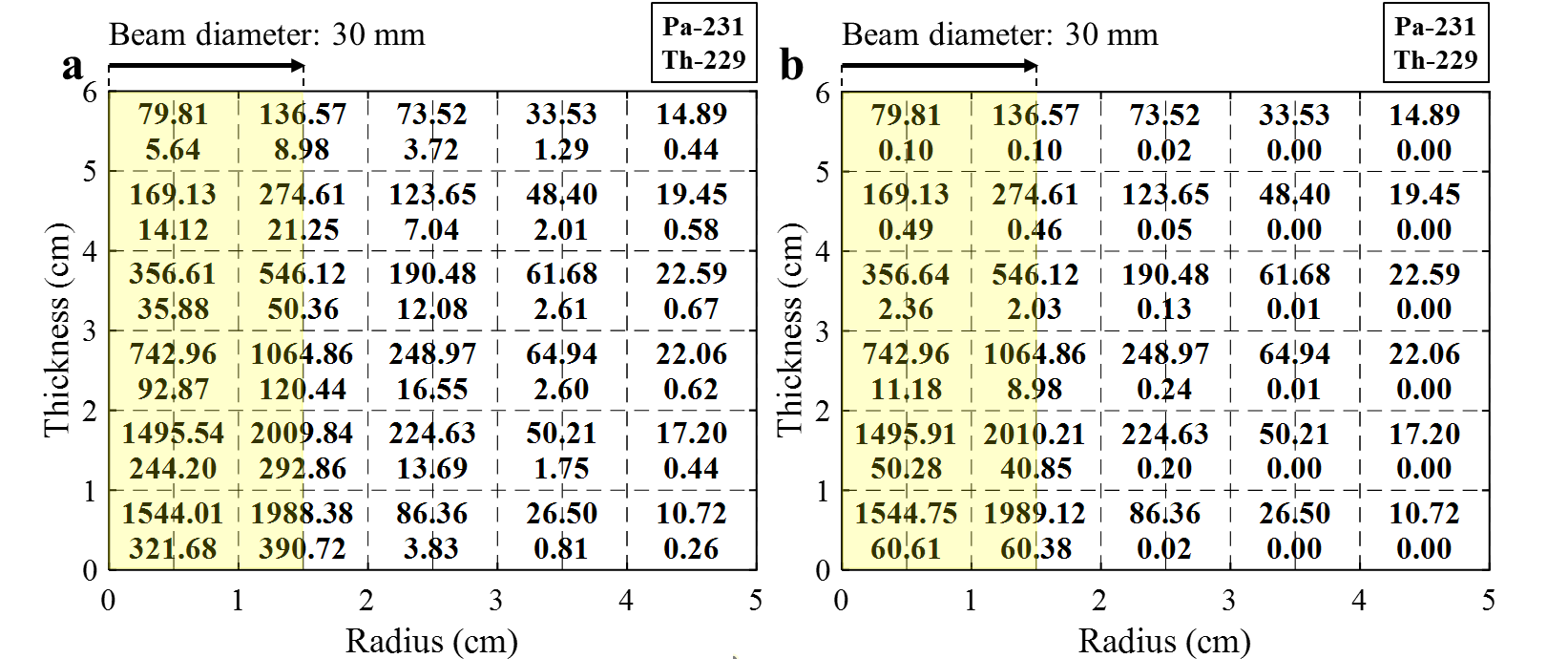


Figure C2. Isotopic yields (MBq) with and without ^232^Th(γ,3n)^229^Th reactions for 30 mm beam diameter


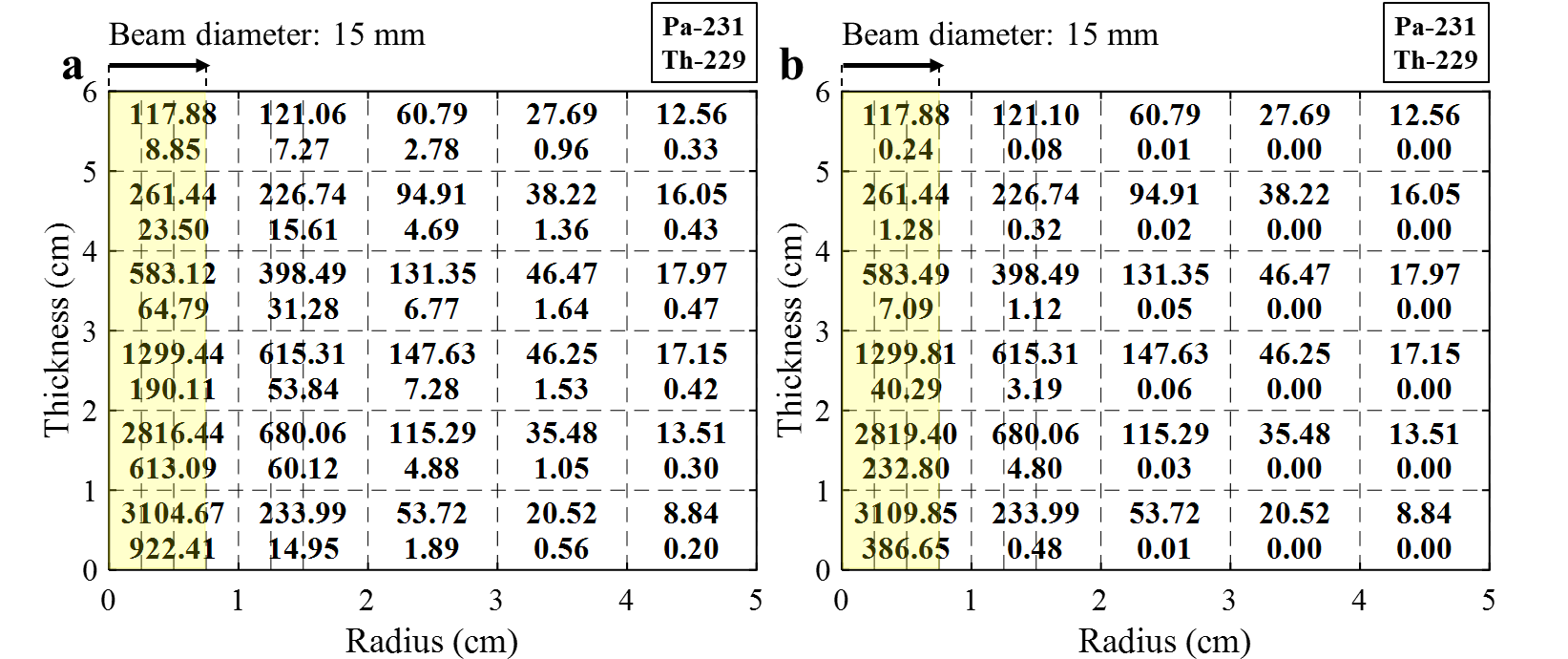


Figure C3. Isotopic yields (MBq) with and without ^232^Th(γ,3n)^229^Th reactions for 15 mm beam diameter

Table C1. Isotopic yields with different electron beam sizes and transmutation pathways

| Diameter (mm)  Yield (MBq) | | 15 | 30 | 40 |
| --- | --- | --- | --- | --- |
| ^229^Th | ^232^Th(γ,3n)^229^Th | 1,365 | 1,432 | 1,446 |
|  | Other pathways  (mainly ^231^Pa(γ,2n)^229^Pa) | 678 | 238 | 148 |
|  | Total yields | 2,043 | 1,670 | 1,593 |
| ^231^Pa | ^232^Th(γ,n)^231^Th | 11,363 | 11,748 | 11,819 |

Most of the ^229^Th yield from the ^231^Pa(γ,2n)^229^Pa reaction is within the irradiated area due to condensed photon distribution as shown in Figs. C1b-C3b. One can see the exponential decrement of the ^229^Th yield from the minor pathways when the diameter is increased from 15 mm to 40 mm in Table C1. It is noted that both yields of ^229^Th and ^231^Pa from the major pathways (^232^Th(γ,3n)^229^Th and ^232^Th(γ,n)^231^Th, respectively) increase with a larger beam diameter. This is because depletion rate of ^232^Th is slower during irradiation in a dispersed electron beam. If the electron beam is completely dispersed, a total ^229^Th yield converges to the yield only due to the ^232^Th(γ,3n)^229^Th reaction. One also notes that, for the 40 mm beam diameter, the total ^229^Th yield only from the ^232^Th(γ,3n)^229^Th reaction is similar to one in Fig. 6 in the main text.

1. **Adjusting ^227^Th milking period**

As discussed in Fig. 9, daily yield of ^227^Th is very low right after a beam irradiation. Consequently, the ^227^Th extraction period should be also affected by a requested minimum yield and it should be quite longer in the early operational period. Figure D1 shows the necessary extraction period to achieve a minimum yield over a long-term operation. For example, the milking period for a minimum yield of 0.111 GBq and 0.185 GBq should be about 19 days and 41 days, respectively, right after irradiation. Table D1 shows the accumulated ^227^Th yield over a 50-year operation with a few minimum yields when the target is irradiated with the electron beam with a diameter of 40 mm. It should be noted that the total accumulated yield is weakly dependent on the minimum required yield.


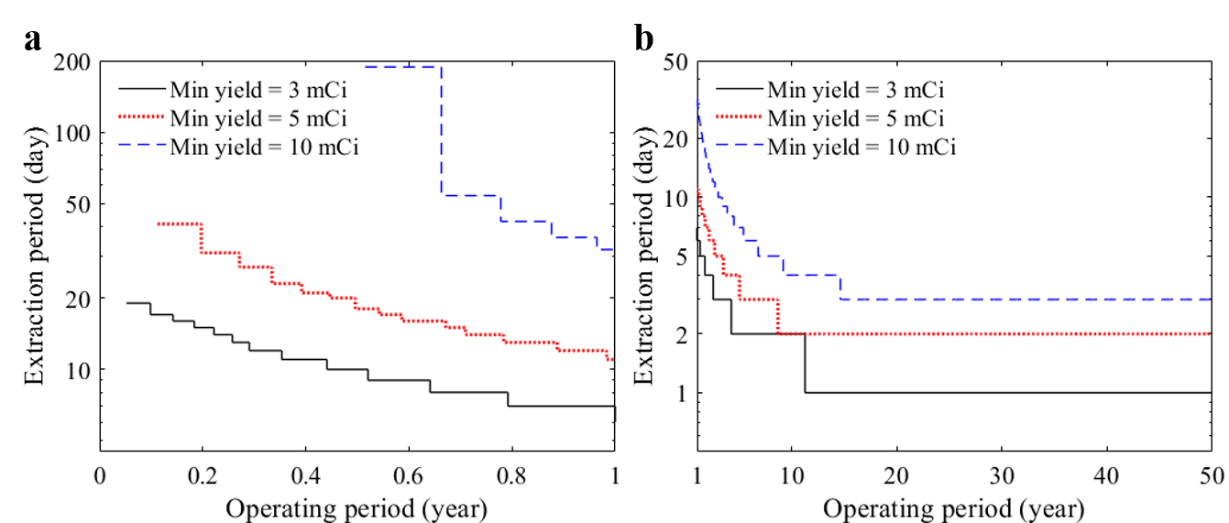


Figure D1. Extraction period vs. operating period with different minimum yields.

Table D1. Accumulated ^227^Th yield over 50 years for several minimum yields per extraction.

| Minimum yield (GBq) | 0.111 | 0.185 | 0.370 |
| --- | --- | --- | --- |
| Accumulated yield (GBq) | 2,458 | 2,411 | 2,353 |

1. **Impacts of TENDL cross-section uncertainty**

Due to limited availability of experimental cross-sections for the photonuclear reactions, the TENDL cross-sections based on the TALYS nuclear model code are used to calculate isotopic yields in this work. The uncertainty of the TENDL data is usually higher than that of experimental ones as TENDL evaluates the data based on both experimental values of similar isotopes and theoretical nuclear models. Figure E1 compares the uncertainty of experimental and TENDL cross-sections of ^232^Th(γ,n)^231^Th and ^232^Th(γ,2n)^230^Th reactions. While most of the experimental data for the (γ,n) reaction are within ±1σ standard deviation of the TENDL data, there is a clear discrepancy for the (γ,2n) reaction between experimental and TENDL data due to difficulties of measuring ^232^Th(γ,2n)^230^Th reaction rate and related parameters. Uncertainty of other Th isotopes is higher than that of ^232^Th as shown in Fig. E2.

Table E1 shows possible variations in the ^225^Ac and ^227^Th yields with the 40 mm electron beam diameter by adjusting TENDL cross-sections of each isotope within ±1σ uncertainty. For example, in order to get a maximum ^225^Ac yield, the TENDL cross-sections of ^232^Th(γ,3n)^229^Th, ^231^Pa(γ,2n)^229^Pa and ^230^Th(γ,n)^229^Th reactions are increased to enhance the ^229^Th production while the removal of ^229^Th is intentionally minimized with reduced TENDL data by 1σ. It is clear that both α-emitter yields are rather sensitive to the TENDL data uncertainty. For the ^227^Th yield, the maximum yield is ~36% higher and the minimum one is also ~36% lower in both 1- and 2-yr irradiations. On the other hand, uncertainty range of the ^225^Ac yield is rather similar in 1-yr irradiation, whereas it is noticeably wider (+51% and -39%) for 2-yr irradiation. This is due to a growing contribution of ^231^Pa(γ,2n)^229^Pa and ^230^Th(γ,n)^229^Th reactions to the ^225^Ac yield and particularly large uncertainty of the ^231^Pa(γ,2n)^229^Pa cross-section.


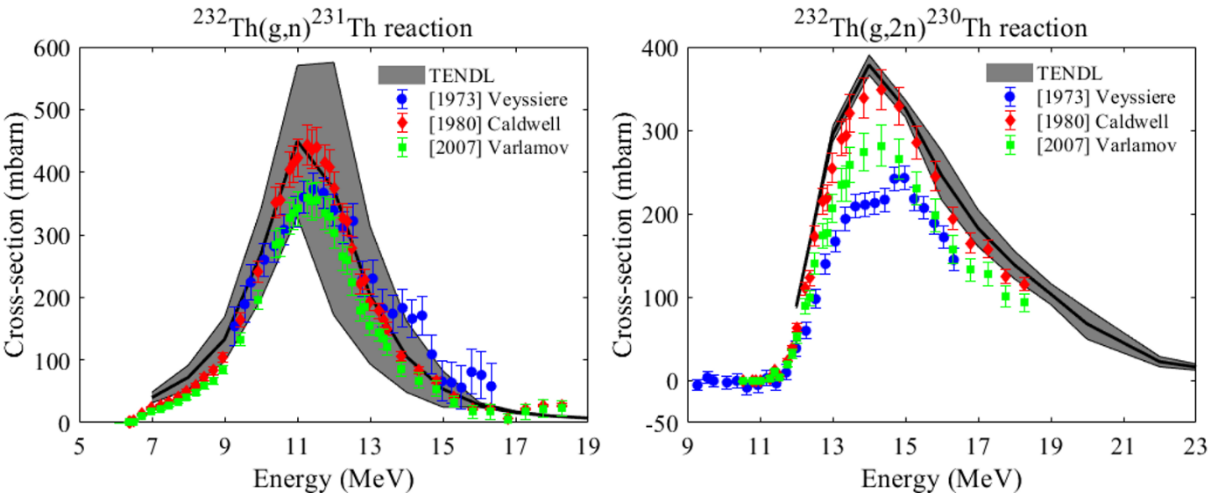


Figure E1. Comparison of experimental cross-sections against TENDL data for ^232^Th(γ,n)^231^Th and ^232^Th(γ,2n)^230^Th reactions.


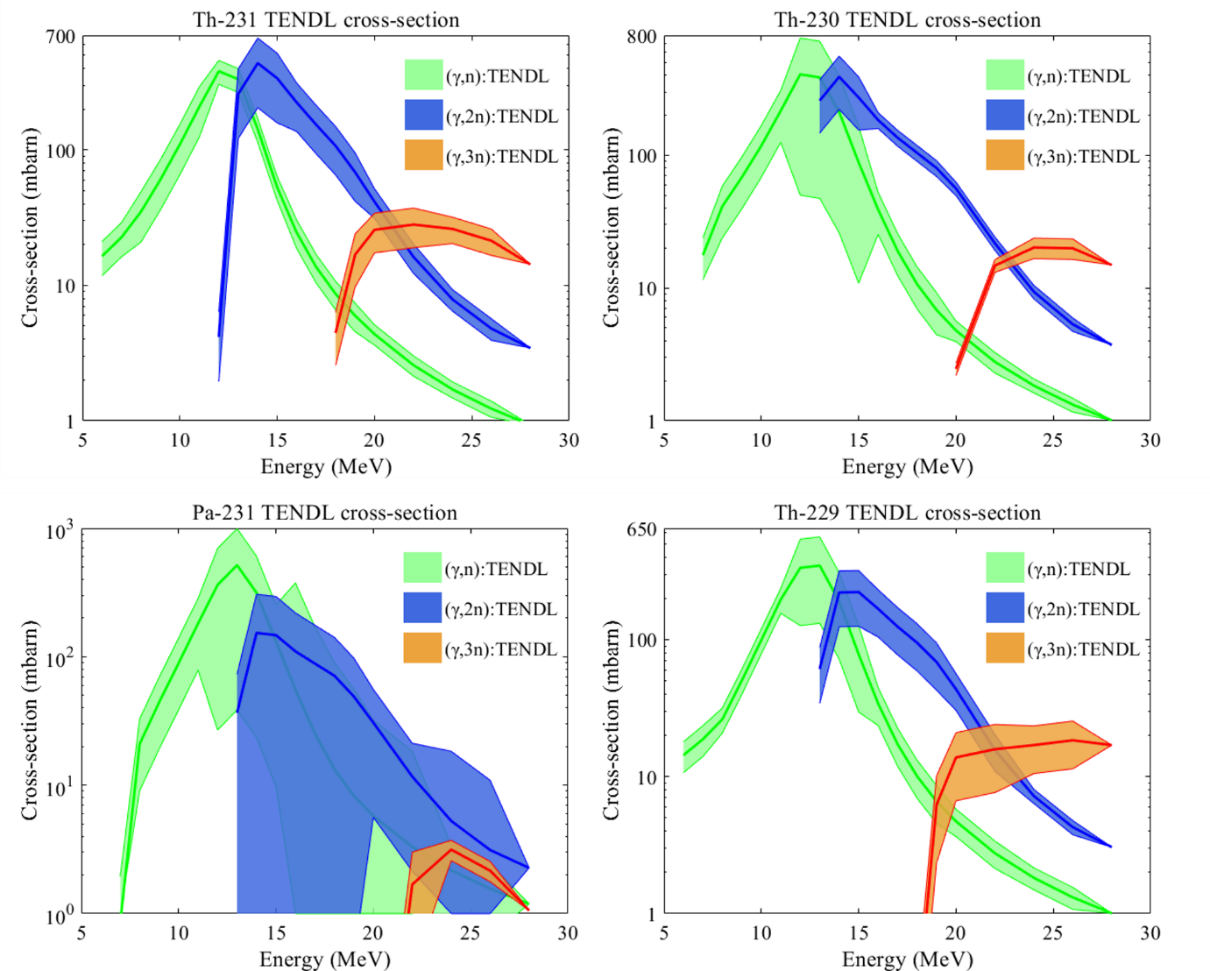


Figure E2. TENDL cross-section and 1σ uncertainty for other isotopes.

Table E1. Yield sensitivity for 1σ adjustment of TENDL cross-section.
(40 mm electron beam diameter, one month cooling and optimal extraction period)

| Yearly yield  (GBq) | 1-yr irradiation | | | 2-yr irradiation | | |
| --- | --- | --- | --- | --- | --- | --- |
|  | Maximum | Mean | Minimum | Maximum | Mean | Minimum |
| ^225^Ac | 11.4 | 8.5 | 6.2 | 25.8 | 17.1 | 10.5 |
| ^227^Th^*^ | 66.6 | 48.9 | 31.5 | 137.2 | 100.1 | 63.9 |

^*^ 50-year average yield

1. **Optimistic ^227^Th yield in the nuclear reactor**

Assuming a ^226^Ra target is irradiated in a nuclear reactor, the optimistic ^227^Th production is evaluated using the following 5-nuclide model:

 Eq. 2-1

 Eq. 2-2

 Eq. 2-3

$\frac{dN^{^{227}Th}}{dt}=\lambda_{Nat}^{^{227}Ac}N^{^{227}Ac}-\lambda_{Nat}^{^{227}Th}N^{^{227}Th},$ Eq. 2-4

$\frac{dN^{^{223}Ra}}{dt}=\lambda_{Nat}^{^{227}Th}N^{^{227}Th}-\lambda_{Nat}^{^{223}Ra}N^{^{223}Ra},$ Eq. 2-5

where *ϕ* is the neutron flux, *σ* is the effective capture cross-section and other notations are standard. All effective cross-sections are taken from EXFOR and E. Kukleva’s work: *σ_226Ra_*=14 barns, *σ_227Ra_*=1 barn and *σ_227Ac_*=800 barns.

In this evaluation, the irradiation time is optimized so that the ^227^Ac yield should be maximized at the end of bombardment (EOB). Table F1 shows dependency of the yearly ^227^Th yield on the neutron flux and target mass. The optimal irradiation time is inversely proportional to neutron flux and the yearly ^227^Th yield is rather proportional to the target mass. One notes that the optimal yield is independent of the neutron flux for a given target mass. It is noteworthy that ^227^Ac activity, source of ^227^Th, should monotonically decrease after EOB in this method.

Table F1. Yearly ^227^Th yield in reactor-based method.

| Mass (g) | 5.135E-6 | 1 | 2 | 5 | 5 | 5 |
| --- | --- | --- | --- | --- | --- | --- |
| Neutron flux (#/cm^2^-sec) | 1.09E+14 | 1.09E+14 | 1.09E+14 | 1.09E+14 | 5.0E+14 | 1.0E+15 |
| Optimal  irradiation (day) | 541 | 540 | 540 | 540 | 119 | 60 |
| ^227^Ac yield at EOB (GBq) | 0.0002 | 43.4 | 86.9 | 217.2 | 219.2 | 219.4 |
| Yearly ^227^Th yield (GBq)^*^ | 0.001 | 288.2 | 576.3 | 1440.7 | 1453.6 | 1455.4 |

^*^50-year average yield

# References

1. Multiphysics, C. Introduction to comsol multiphysics®. *COMSOL Multiphysics, Burlington, MA, accessed Feb* **9**, 2018 (1998).
